# Prevalence of Balance Impairment and Factors Associated with Balance among Patients with Stroke. A Cross Sectional Retrospective Case Control Study

**DOI:** 10.3390/healthcare9030320

**Published:** 2021-03-13

**Authors:** Fayaz Khan, Mohamed Faisal Chevidikunnan

**Affiliations:** Department of Physical Therapy, Faculty of Medical Rehabilitation Sciences, King Abdulaziz University, Jeddah 21589, Saudi Arabia; mfaisal@kau.edu.sa

**Keywords:** stroke, balance, prevalence, risk factors, impairment

## Abstract

Stroke is a major cause of disability worldwide, and balance impairments are common disabling factors in patients with stroke, leading to falls. Thus, the study objectives were as follows: (i) To find the prevalence of balance impairment among patients with stroke. (ii) To find out the factors associated with balance impairment in patients with stroke. This cross-sectional retrospective case control study involved eighty-one post stroke patients with a mean age of 58.36 ± 14.06, recruited from six hospitals, who underwent an assessment of balance, walking speed, depression and isometric strength of the ankle and knee. These patients were later categorized into subjects with good balance (<45) in the Berg balance scale (BBS) and those with poor balance (≥45), as cases and controls, to assess the factors associated with balance impairment using binary logistic regression. The prevalence of balance impairment among patients with stroke was 48.1%. The reduction in power of knee flexors (OR = 0.858), knee extensors (OR = 0.880) and ankle dorsiflexors (OR = 0.820) was found to be significantly associated with balance impairment, along with speed (OR = 1.187 (95% CI = 1.100, 1.280)), depression (OR = 1.331 (95% CI = 1.055–1.679)) and activities of daily living (OR = 0.313 (95% CI = 0.150–0.650)). In summary, around half of the patients with stroke exhibited balance impairments, with females being more prone.

## 1. Introduction

Stroke is one of the leading causes of mortality worldwide [1], and is identified as one of the most common causes of disability [2]. Stroke patients face challenges such as motor, sensory and communication deficits, and in areas such as cognition [3,4,5,6], quality of life [7,8,9], and mental and physical health [10,11]. The impacts of these impairments are reflected in the survivors in many ways, one of which is the fact that some stroke survivors often experience social isolation, which could lead to post-stroke depression and anxiety [12,13,14]. Balance impairment is another challenge post-stroke patients face. This has been identified consistently in many studies as a risk factor for falls and fear of falling [15,16,17,18,19]. As such, fall-related injuries are expected to occur, further increasing the rate of mortality among stroke survivors [20].

Given the negative consequences of balance impairments in patients with stroke, it is thus critical to know the prevalence of balance impairment. Exploring the risk factors that are associated with balance impairment in patients with stroke can enable their easy identification, leading to prompt care.

Multiple reports have been issued thus far on the prevalence of balance impairments post-stroke, with values ranging from 16.7% to as high as 83% [21,22]. Difference in these values are most probably caused by differences between the date of onset and the date of assessment, assuming acute patients have worse balance than chronic patients. The mean age of participants in each study is also another probable cause, as balance function has been shown to diminish with age [23]. Gender, handedness, location and side of lesion are all factors affecting balance, which are explored in the literature along with stroke severity. Assessment of balance at admission could explain the motor response to the rehabilitation programs in subacute patients, and could also act as a predictor for balance during the discharge [24]. Similarly, balance confidence has been reported to predict perceived physical function, mobility and recovery at 12 months in patients with stroke [25]. Good balance is also important for stepping patterns, gait velocity, and initial training of gait in subjects with stroke [26,27]. Moreover, cognitive function also affects balance, and it has been reported that patients with cognitive dysfunction had balance impairments and a higher risk of falls.

Data on stroke in Saudi Arabia are evidently lacking, and this marked lack of evidence in the region regarding both the incidence and the prevalence of stroke has made the assessment of prevalence of post-stroke balance impairments even more difficult [28]. With the importance of establishing the prevalence of balance impairments and the factors associated, it is accordingly necessary to provide such a study for the region. Thus, the aim for this study was to find the prevalence of balance impairment and the factors associated with it in patients with stroke

## 2. Materials and Methods

This cross-sectional retrospective case control study involved eighty-one subjects with stroke, and the sample was determined according to Kesley’s method, keeping the power at 80 and with a 95% CI. The subjects was recruited from four hospitals in Jeddah, Saudi Arabia. The inclusion criteria included the following: (a) patients who received a diagnosis of ischemic brain injury, or intracerebral or subarachnoid hemorrhage, by MRI or CT, with duration of 2 weeks or more since the onset of stroke; (b) patients who walk independently for 5–10 m with or without an assistive device; (c) patients who can follow simple instructions and sign the consent form; (d) patients who are 18 years of age and older. The following patients were excluded: (a) patients with orthopedic, cardiovascular, neurological or pulmonary conditions that may restrict assessment; (b) medically unstable patients; (c) patients with bilateral strokes; (d) patients with pre-stroke balance impairments; (e) ataxic patients.

The general data included gender, age, type of stroke, side of weakness and time since stroke. The patients were also assessed for variables including balance, via the Berg balance scale (BBS). The BBS is a 14-item scale, requiring 10 to 20 min to complete and measure the patient’s balance, either statically or while preforming various functional movements. The items are scored from 0 to 4, with the score of 0 as an inability to complete the task and a score of 4 representing independent item completion. The muscle strength of knee flexors and extensors, as well as the strength of ankle dorsiflexors and plantar flexors, were measured using a handheld dynamometer (HHD). Speed was assessed via a timed ap and go (TUG) test, wherein the patient is timed while he/she rises from a chair with armrests and walks at a comfortable and safe pace to a marked point 3 meters away, then turns and walks back to sit down again. Activities of daily living were assessed using the Barthel index (BI), which measures the extent to which somebody can function independently and has mobility in their activities of daily living (ADL), i.e., feeding, bathing, grooming, dressing, bowel control, bladder control, toileting, chair transfer, ambulation and stair climbing. Level of depression was assessed via the geriatric depression scale (GDS), which is a screening test used to identify symptoms of depression in older adults, and the use of walking aids was also noted.

Informed consent was obtained from the participants before they were enrolled in the study; ethical approval was obtained from the Centre of Excellence in Genomic Medical Research (04-CEGMR-Bioeth-2019), approved by the National Committee of Bioethics (KACST: HA-02-J-003).

### Analysis

The data were analyzed using statistical software SPSS version 21 (SPSS, Inc., Chicago, IL, USA) and Graph Pad Prism version 6.0 (GraphPad Software Inc., La Jolla, CA, USA). Mean, standard deviation, percentages, median, and range were used to describe the different characteristics of the participants. The prevalence of balance impairment was presented as a percentage. The significance of cases and controls is represented as odds ratio and *p* value at *p* ≤ 0.05.

## 3. Results

Out of the 116 eligible subjects, 81 were included, with a response rate of 70%. The age of participants ranged from 25 to 94 years, with a mean of 58.36 ± 14.06. The basic characteristics of the study subjects are mentioned in Table 1. The mean BBS scores were 39.94 ± 13.76, and 39 (48.1%) of the stroke survivors exhibited balance impairment.

### Regression Analysis

A simple binary logistic regression analysis (Enter) method was done, keeping balance as the dependent variable to predict the influence of different independent variables. Odds ratio (OR) was used to find the influence of variables, keeping one of the dichotomous variables as the reference value. For the purpose of binary logistic regression, different variables were categorized dichotomously. (i) Balance; good balance (<45) and poor balance (≥45). (ii) Age; < 55 and ≥ 56. (iii) Gender; male and female. (iv) Side; left and right. (v) Type of stroke; ischemic and hemorrhagic. (vi) Duration; acute/subacute (<6 months of onset) and chronic (>6 months of onset). (vii) Aid; with aid and without aid. Cross-tabulation with a bivariate analysis was done to assess the effect of each variable on balance impairment, and significance was determined via chi square.

**Figure 1:**
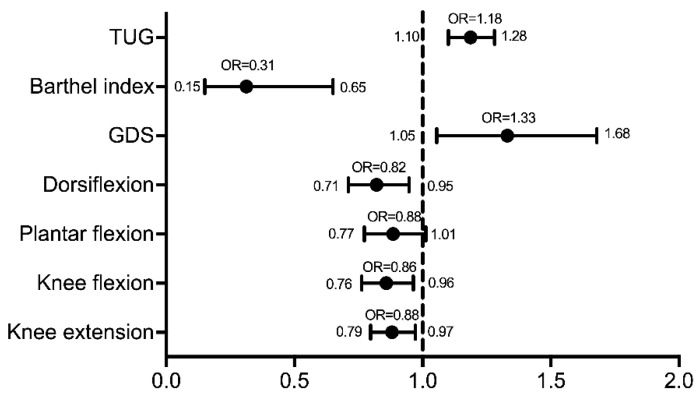
Odds ratio with 95% CI for different variables affecting balance.

The results of the binary logistic regression analysis showed that use of a walking aid, knee flexion and extension strength, ankle dorsiflexion strength, depression, speed and level of ADL are the variables that are significantly associated with balance impairment. Participants requiring a walking aid had a higher prevalence of balance impairment (77.8%), and were ten times more likely to have their balance impaired post-stroke in comparison to non-aid requiring patients (odds ratio (OR) = 10.8 (95% CI = 3.827–30.577)) (Table 2). The strength of dorsiflexors (OR = 0.820 (95% CI = 0.710–0.947)), knee flexors (OR = 0.858 (95% CI = 0.763–0.964)) and knee extensors (OR = 0.880 (95% CI = 0.797–0.972)) were all factors that increased the risk of balance impairment in patients after stroke, which has been illustrated in Figure 1, showing the odds ratio and 95% confidence interval of each variable. Speed was another variable that affected balance; with every increment in TUG score, the patient becomes more susceptible to having their balance affected (OR = 1.187 (95% CI = 1.100–1.2800). Similarly, for every increase in depression, the patient’s risk of balance impairment also increases (OR = 1.331 (95% CI = 1.055–1.679)). When the level of ADL decreases, the risk of balance impairment was also shown to increase (OR = 0.313 (95% CI = 0.150–0.650)).

**Figure 2:**
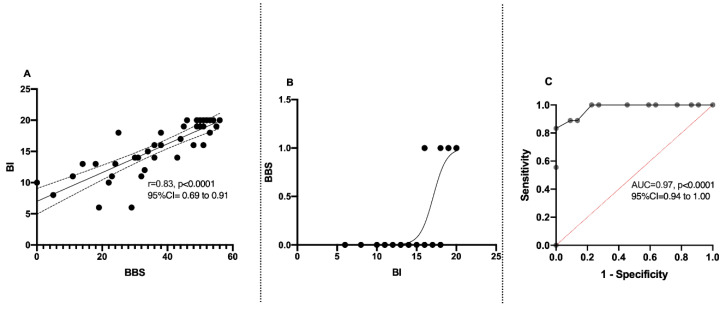
Relation between balance (BBS) and Barthel index (BI): (**A**) correlation between BBS and BI; (**B**) regression curve on BBS and BI; (**C**) ROC curve between BBS and BI.

Those factors found to be significant upon univariate analysis (*p* ≤ 0.05) were further entered into logistic regression models with a forward stepwise (likelihood ratio) method, in which the probability of the entry of the variables was fixed as 0.05 and that of their removal as 0.10. Activities of daily living measured by the Barthel index was the only factor influencing balance impairment in the model (Figure 2). Furthermore, the BBS scores were correlated with the BI, showing an excellent correlation of r = 0.83 (*p* < 0.0001; 95% CI = 0.69 to 0.91) and good sensitivity and specificity on the receiver operating curve, with an excellent area under the curve value of 0.97 (*p* < 0.0001, 95% CI = 0.94 to 1.00).

The goodness of fit assessed via the Hosmer and Lemeshow tests showed *p* = 0.79, χ^2^ = 3.09, with an overall 90% predictable variance in the one-step model.

## 4. Discussion

The study assessed the prevalence of balance impairment in patients with stroke and the factors associated with balance impairment in four hospitals in Saudi Arabia. The assessment of balance in patients with stroke should be considered with importance, as it is a significant predictor of long-term physical function, mobility and perceived recovery. The results of the current study are expected to be of help to researchers and clinicians in evaluating and planning effective treatment strategies. The existing data available are from western countries [21], subcontinents [29], and Africa [22]; however, the data from the Arab population were deficient, thus making their future study necessary.

The mean age of the subjects with stroke in this study was 58.36 ± 14.06; however, the mean ages of stroke subjects in studies from developed countries were higher, while those from the subcontinent were lower, and those from Africa were similar to the results in this study [21,22,29]. Nevertheless, these distinctions underpin the desideratum of prevalence data from these kinds of studies.

The prevalence of balance impairment in this study was 48.1%, indicating that the impairment was present in half of the stroke survivors. The data on prevalence reported from previous studies were in contrast to this study’s findings. The results from a previous study in the United Kingdom reported a high prevalence of balance (83%) [21]. It is important to note that the study was conducted in the acute period, in which the mean duration was 21 ± 5 days, which could have resulted in such a high prevalence. However, the studies from Nigeria (36.8%) [22] and Sri Lanka (16.7%) [29] reported a lower prevalence in balance impairments. These results were very much related to the age of the population, affirming that the elder population have a greater prevalence of balance impairment. It would be advantageous to include a balance assessment in the routine physical therapy protocol for the planning and implementation of treatment. For instance, a survey conducted in Saudi Arabia on the perspectives of physical therapists on patients with stroke found that they employed standardized assessment using the Fugl Meyer scale; however, the balance assessment was excluded [30].

The current study has demonstrated that the stroke-related factors had a more significant relation with balance impairment than the sociodemographic factors. However, a variable relation between balance impairment and different stroke-related factors is noted in previous studies [21,31,32], while on the contrary, stroke-related factors, such as severity [21] and side of stroke [32], have been found to be significantly related to balance impairments.

Gender differences showed that males were less likely to have balance impairments when compared to their female counterparts [22]. Among the stroke-related factors, acute and subacute stroke survivors are 1.5 times more prone to develop balance impairment when compared to chronic patients. This finding was supported by a previous study [22], suggesting that in the chronic phase, a substantial amount of spontaneous recovery might have already happened; furthermore, the rehabilitation therapies that would have been administered in the acute phase might have translated their effects into the chronic phase.

In this study, the variables that have a significant relation with balance impairment are use of a walking aid, speed, knee flexion and extension strength, ankle dorsiflexion strength, depression, ADL and disability. These are considered to be modifiable factors; however, in previous studies, the factors that significantly influenced balance were mostly non-modifiable factors, such as age, gender, and post-stroke duration [22].

The association of stroke with depression has been established in the literature, with almost 20% of stroke survivors experiencing depression in the acute stage [33]. This, when combined with the findings of this study, implies that depression further increases the risk of balance impairment. In this instance, assessing depression and balance [34] in patients with stroke is thus essential, along with ADL and disability level. Such assessments should be routinely administered by healthcare practitioners in order to provide the proper treatment and care. The integration and use of a multidisciplinary team in the treatment and rehabilitation of stroke is thus emphasized by these findings [35]. In addition, the use of workplace physical activity can be integrated into the rehabilitation of patients with stroke [36]. Although less explored in literature, the strength of lower limb muscles and their ability to predict balance impairment post-stroke is another important finding that could be easily incorporated into a physical therapy rehabilitation program for stroke patients in order to improve balance [37].

The limitations in this study include the male to female (3:1) ratio, with male participants being the larger group. The total sample size was also small for a prevalence study, due to the lack of information regarding the prevalence of stroke in the region, making the estimation of the proper sample size of the study difficult. Further case control studies are warranted with an age-matched population in order to address the ratio of difference between the controls and patients with stroke. In particular, longitudinal studies would be beneficial for addressing the long-term outcomes of balance impairment, the factors affecting it, and the influence of rehabilitation strategies on balance.

## 5. Conclusions

Around half of the patients with stroke also exhibit balance impairments, with females having a greater incidence. Speed, depression, level of ADL, use of a walking aid, and the strength of ankle dorsiflexion, knee flexion, and knee extension were all found to be significant factors influencing balance in patients with stroke. As such, all these relations should be taken into consideration when planning rehabilitation in patients with stroke.

## Figures and Tables

**Table 1:** Characteristics of the participants (n = 81).

Variables	Value
	n (n%)
Gender	
Male	64 (79%)
Female	17 (21%)
Side of Weakness	
Right	34 (42%)
Left	47 (58%)
	Mean ± SD (range)
Age (years)	58.36 ± 14.06 (25 to 94)
Time since stroke (Months)	16.96 ± 32.43 (1 to 223)
Ankle dorsiflexion strength (kg)	3.97 ± 3.76 (0 to 18)
Ankle plantarflexion strength (kg)	5.04 ± 3.41 (0 to 14)
Knee extension strength (kg)	9.51 ± 5.44 (0 to 30)
Knee flexion strength (kg)	5.94 ± 4.57 (0 to 20)
TUG (second)	34.50 ± 27.28 (6.750 to 165.0)
	Mean ± SD; Median (range)
BBS	39.94 ± 13.76; 45 (0 to 56)
BI	15.63 ± 4.19; 16 (6 to 20)
GDS	4.725 ± 3.52; 4.500 (0 to 13)
mRS	2.575 ± 1.28; 2 (1 to 5)

**Table 2:** Prevalence of balance for different variables and factors affecting balance.

Variable	Prevalence of Balance (%)	*p* Value	Odds Ratio	95% CI
Gender				
Female	52.9	0.657	1.28	0.437–3.723
Male	46.9	-	1	Reference
Age (Years)				
≥56	50	0.702	1.19	0.492–2.866
<55	45.7	-	1	Reference
Post stroke duration				
Acute/subacute	53.8	0.324	1.556	0.647–3.741
Chronic	42.9	-	1	Reference
Type of stroke				
Ischemic	53.3	0.855	1.143	0.273–4.786
Hemorrhagic	50	-	1	Reference
Side of Hemiplegia				
Left	51.1	0.537	1.322	0.545–3.206
Right	44.1	-	1	Reference
Aid				
With aid *	77.8	0.0001	10.818	3.827–30.577
Without aid	24.4	-	1	Reference

* Significant.

## Data Availability

The data presented in this study are available on request from the corresponding author. The data are not publicly available due to the regulations from funding agency.

## Abbreviations

TUG	Timed up and go test
BBS	Berg balance scale
BI	Barthel index
GDS	Geriatric depression scale
mRS	modified Rankin scale
